# Statin treatment affects cytokine release and phagocytic activity in primary cultured microglia through two separable mechanisms

**DOI:** 10.1186/s13041-014-0085-7

**Published:** 2014-11-26

**Authors:** Matthew A Churchward, Kathryn G Todd

**Affiliations:** Neurochemical Research Unit, University of Alberta Faculty of Medicine, Edmonton, AB Canada T6G 2R3; Department of Psychiatry, University of Alberta Faculty of Medicine, Edmonton, AB Canada T6G 2R3; Neuroscience and Mental Health Institute, University of Alberta Faculty of Medicine, Edmonton, AB Canada T6G 2R3

**Keywords:** Inflammation, Cholesterol, Mevalonate, Phagocytosis

## Abstract

**Background:**

As the primary immune cells of the central nervous system, microglia contribute to development, homeostasis, and plasticity of the central nervous system, in addition to their well characterized roles in the foreign body and inflammatory responses. Increasingly, inappropriate activation of microglia is being reported as a component of inflammation in neurodegenerative and neuropsychiatric disorders. The statin class of cholesterol-lowering drugs have been observed to have anti-inflammatory and protective effects in both neurodegenerative diseases and ischemic stroke, and are suggested to act by attenuating microglial activity.

**Results:**

We sought to investigate the effects of simvastatin treatment on the secretory profile and phagocytic activity of primary cultured rat microglia, and to dissect the mechanism of action of simvastatin on microglial activity. Simvastatin treatment altered the release of cytokines and trophic factors from microglia, including interleukin-1-β, tumour necrosis factor-α, and brain derived neurotrophic factor in a cholesterol-dependent manner. Conversely, simvastatin inhibited phagocytosis in microglia in a cholesterol-independent manner.

**Conclusions:**

The disparity in cholesterol dependence of cytokine release and phagocytosis suggests the two effects occur through distinct molecular mechanisms. These two pathways may provide an opportunity for further refinement of pharmacotherapies for neuroinflammatory, neurodegenerative, and neuropsychiatric disorders.

## Background

Microglia, the innate immune cells of the central nervous system (CNS), carry out surveillance and are activated in response to foreign bodies, infectious agents, and local damage. On activation, microglia exhibit a spectrum of behaviours including initiation of the inflammatory response, migration, proliferation, and phagocytosis of foreign particles and cellular debris, and modulation of the microenvironment through synthesis and release of trophic and toxic effectors. These curious cells are, however, far from quiescent in their ‘resting’, ramified, or surveilling state, playing roles in development and maintenance of the CNS including population control of neuronal precursor cells, modulating neuronal plasticity through synaptic pruning, and maintaining homeostasis in concert with other resident glial cells [1–7]. Unfortunately, inflammation mediated by microglia has been implicated in an increasing number of pathological conditions ranging from acute injuries such as ischemic or hemorrhagic stroke [8] and traumatic brain or spinal cord injury [9], chronic inflammatory conditions such as multiple sclerosis [10,11], neurodegenerative diseases including Alzheimer’s [12], Parkinson’s [13], and vascular dementia [14], and even neuropsychiatric disorders including major depressive disorder [7,15,16], schizophrenia [17–19], and bipolar disorder [20]. The cumulative disease burden of neuroinflammatory conditions involving microglia is substantial, and, as many of these pathologies have unknown or poorly understood etiology, targeting the common inflammatory component represents a viable approach to improving immediate health outcomes [21].

The statin class of 3-hydroxy-3-methylglutaryl-coenzyme A (HMG-CoA) reductase inhibitors have been one of the most widely prescribed and successful classes of drugs of all time. While statins were characterized for their inhibition of cholesterol biosynthesis and lowering of blood cholesterol levels, subsequent mechanistic studies have suggested much of their action in reducing risks for cardiovascular disease are unrelated to cholesterol levels, relating rather to pleiotropic effects including isoprenoid biosynthesis and subsequent protein prenylation [22,23]. Studies of blood derived monocytes and macrophages have highlighted a number of anti-inflammatory effects of statins including altered cytokine release and phagocytic activity [24–29]. In the CNS statin treatment has been suggested to delay the onset and slow the progression of Alzheimer’s disease and dementia [30–32], and statin treatment after ischemic stroke and thrombolysis has recently been shown to improve functional outcomes and reduce risk of neurological deterioration and death [33]. Studies in rodents have shown statin treatment improved outcomes in models of ischemic stroke [34,35], intracerebral hemorrhage [36], traumatic brain injury [37], and Alzheimer’s disease [38,39]. Despite a wealth of evidence that statins directly modulate macrophage function [24–29] and observation of changes in microglial and inflammatory markers in the CNS after statin treatment [35–39] studies of the direct effects of statin treatment on microglia have been sparing, and present conflicting data. In primary rodent microglial cultures, lovastatin decreased release of inflammatory mediators nitric oxide (NO), tumour necrosis factor-α (TNFα), and interleukin-1β (Il1β) [40] and simvastatin treatment decreased surface antigen expression including major histocompatibility complex II (MHCII) and chemokine receptor CXCR3 [41] while in primate microglial cultures simvastatin increased secretion of cytokines (TNFα, Il1β, interleukin-12, and interleukin-6) [42] and in rat hippocampal slice cultures mevastatin treatment increased cytokine release and surface antigen expression (TNFα, cluster of differentiation[CD]-11b) [43]. In the current study, we sought to analyse the direct effects and mechanism of statin treatment on primary cultured microglia. We demonstrate for the first time that simvastatin treatment inhibits microglial phagocytosis and alters the release of inflammatory mediators and growth factors via two independent and separable molecular mechanisms.

## Results

### Simvastatin affects microglial secretion through cholesterol-dependent mechanisms

**Figure 1:**
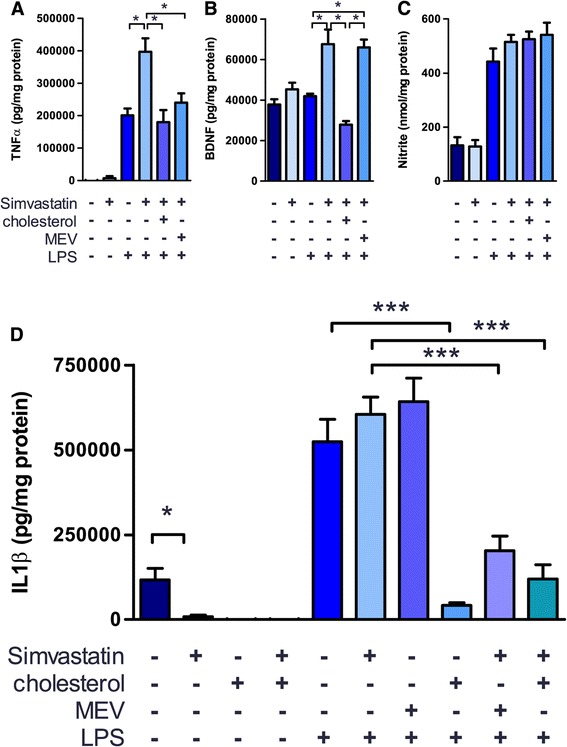
**Statin treatment altered the secretory profile of microglia.** Isolated rat microglial cultures were treated with 20 μM simvastatin for 24 h and subsequently activated with 1 μg/ml LPS overnight (18 h). For rescue experiments microglia were concurrently treated with 200 μM MEV for 24 h or subsequently treated with 1 mM hpβcd-cholesterol 30 minutes prior to LPS stimulus. Media was collected and assayed for cytokine release (Il1β and TNFα, **A**,**D**), neurotrophin release (BDNF, **B**), and nitric oxide secretion **(C)**. All analyses were normalized to the total protein of the corresponding cell lysate to control for cell viability. Asterisks represent P <0.05, 0.01, and 0.001 by 2-way ANOVA and Bonferroni’s post-hoc from N = 6 independent preparations.

Initial dose response experiments were performed to determine the efficacy of simvastatin (from 1 μM to 80 μM) at reducing microglial cholesterol, and showed 20 μM to consistently reduce cellular cholesterol levels without adverse effects on cell viability (data not shown). This dose is consistent with comparable studies in the literature [43–46]. Treatment of primary microglia with 20 μM simvastatin (Figure 1A-D), which decreased mean cellular cholesterol by 18.0 ± 3.6%, was found to alter the release of a number of microglial mediators into culture media, including interleukin-1β (Il1β), tumour necrosis factor-α (TNFα), and brain-derived neurotrophic factor (BDNF). TNFα release was not detectable in microglia prior to activation with LPS, however simvastatin treatment significantly increased the release of TNFα after LPS treatment (Figure 1A). BDNF release was similarly affected by simvastatin: treatment increased the release of BDNF selectively from LPS-activated microglia (Figure 1B). Interestingly, simvastatin did not have a significant effect on the release of NO from microglia (Figure 1C), and LPS-activated microglia showed comparable increases in NO release in all treatment groups. Simvastatin treatment significantly decreased the basal release of IL1β from microglia (Figure 1D), however IL1β release after exposure to a potent inflammatory stimulus, bacterial lipopolysaccharide (LPS), was not significantly different between statin treatment and control (Figure 1D). Treatment with simvastatin at the doses reported had no effect on the viability of microglia as assessed using an MTT assay (data not shown). While the overall effects of simvastatin treatment on cytokine and trophic factor release are mixed and dependent on activation state, with decreased Il1β release from unstimulated microglia and increased TNFα and BDNF release from LPS-activated microglia, these data warranted further investigation into the mechanism of statin-mediated changes in secretory profile.

As statins have been proposed to affect both cholesterol-dependent and cholesterol-independent (principally through alterations in isoprenoid biosynthesis) cellular processes, simvastatin treated microglia were supplemented with cholesterol, solubilized in complex with 2-hydroxypropyl-β-cyclodextrin (hpβcd-cholesterol), or mevalonolactone (MEV), the lactone isomer of mevalonic acid, the immediate product of HMG-CoA reductase. Supplementation of cholesterol is expected to affect only cholesterol-dependent processes, while MEV supplementation is expected to affect both cholesterol-dependent and independent processes. The effects of simvastatin treatment on TNFα and BDNF release were consistently reversed by treatment with hpβcd-cholesterol: TNFα release from LPS-activated microglia was decreased to that of the control when cells were treated with simvastatin followed by either hpβcd-cholesterol or MEV (Figure 1A), while BDNF release was similarly decreased to the level of control after treatment with simvastatin and hpβcd-cholesterol, but not MEV (Figure 1B). Release of Il1β was particularly sensitive to cholesterol, as hpβcd-cholesterol treatment alone was able to decrease Il1β release in both unstimulated and LPS-activated microglia (Figure 1D). Co-treatment of LPS-activated microglia with simvastatin and MEV was likewise able to decrease Il1β release from LPS-activated microglia, but MEV treatment in LPS-activated microglia alone did not affect Il1β release. While these findings are confounded by the effects of cholesterol alone on Il1β release, they are suggestive of a cholesterol-dependent effect of simvastatin treatment.

### Simvastatin inhibits microglial phagocytosis through a cholesterol-independent mechanism

**Figure 2:**
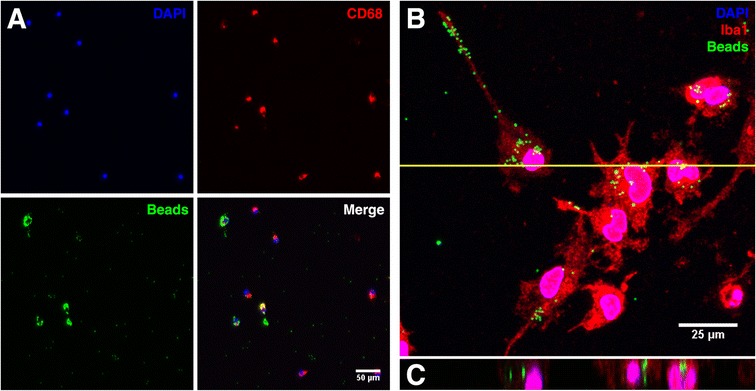
**Immunofluorescent measurement of phagocytosis. A** – Representative image of microglia incubated with fluorescent latex beads (green), immunolabelled for CD68 to identify the cell soma (red) and nuclei stained with DAPI (blue), scale bar represents 50 μm. **B** – Representative confocal micrograph to demonstrate bead internalization. Microglia were incubated with fluorescent latex beads (green), immunolabelled for Iba1 to visualize cytoplasmic volume (red), and nuclei stained with DAPI. Scale bar represents 25 μm, yellow line indicates the region projected orthogonally in **C**.

Phagocytosis was assayed by immunofluorescent microscopy of microglia previously incubated with green fluorescent carboxylate-modified latex beads [47] (Figure 2A). To validate bead internalization in our assay system microglia were processed for immunofluorescence using the microglia-specific cytosolic marker ionized Ca^2+^-binding protein-1 (Iba1), effectively labelling the total cell volume (Figure 2B). A series of confocal z-stacks were acquired and projected orthogonally to demonstrate that beads were present within the cytosolic compartment (Figure 2C) rather than simply adhering to the cell surface.

**Figure 3:**
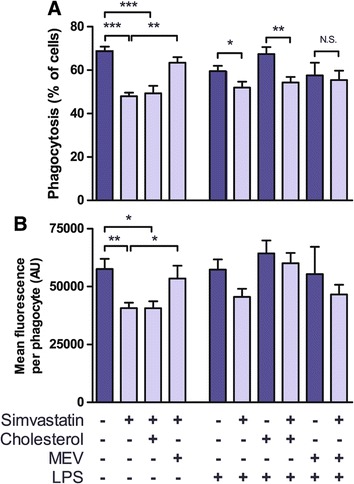
**Simvastatin treatment and cholesterol depletion reversibly inhibit microglial phagocytosis.** Isolated rat microglial cultures were treated with simvastatin (**A**-**B**, treatments as in Figure 1). 200 μM MEV was added concurrently for 24 h, while 1 mM hpβcd-cholesterol was added for 30 min at the end of the 24 h simvastatin treatment. At the end of treatment cells were incubated with carboxylate-modified green fluorescent latex beads (1x10^7^/ml) for 3 h, fixed and immunolabelled for the microglia-specific antigen CD68. Phagocytosis was quantified as the proportion of cells having taken up at least 1 bead (proportion of microglia involved in phagocytosis; **A)** and as the mean fluorescence per cell having taken up at least 1 bead (mean uptake per active phagocyte; **B)**. Asterisks represent P <0.05, 0.01, and 0.001 by 2-way ANOVA with Bonferroni’s post-hoc for N = 6 **(A-B)** independent culture preparations.

Simvastatin treatment significantly decreased phagocytosis in both the proportion of cells taking up latex beads as well as the mean uptake per active phagocyte (Figure 3A-B). This effect was observed in both unstimulated microglia and after LPS-activation, though the effect was most pronounced in the unstimulated group, and the difference in mean fluorescence did not reach significance for the LPS-treated group. As with previous assays of cytokine and trophic factor release, we recovered cholesterol directly with hpβcd-cholesterol to examine cholesterol-dependent contributions, and used co-treatment with MEV to examine cholesterol-independent contributions. The proportion of active phagocytes remained significantly suppressed in cells treated with simvastatin followed by hpβcd-cholesterol without LPS stimulus, and remained significantly decreased relative to the cholesterol control group in LPS-activated microglia (Figure 3A). Co-treatment with MEV resulted in a significant increase in active phagocytes and mean uptake per cell relative to the simvastatin treated group without LPS stimulus (Figure 3A-B), and after LPS-activation MEV co-treatment abolished the significant difference from the respective control condition (but did not reach significance from either LPS-only control or simvastatin + LPS groups). No significant differences were observed in mean uptake per cell in the presence of LPS (Figure 3B). These data suggest that in the presence or absence of an LPS stimulus microglial phagocytosis is sensitive to inhibition by simvastatin through cholesterol-independent mechanisms.

### Direct cholesterol manipulation affects microglial secretion and phagocytosis

**Figure 4:**
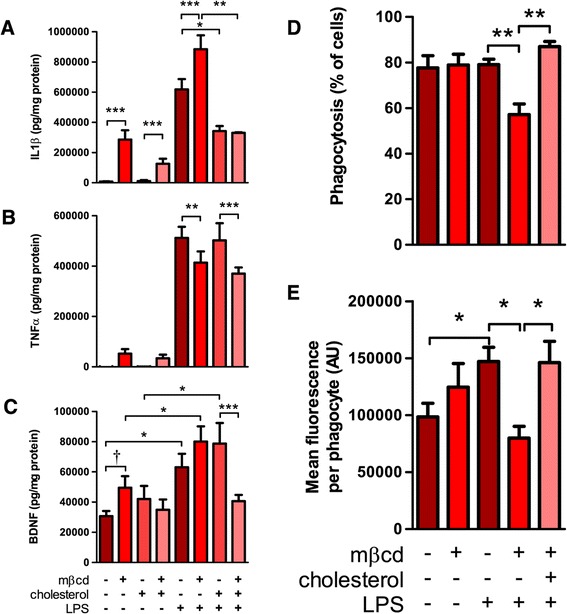
**Cholesterol depletion and augmentation altered both phagocytosis and the release of Il1β, TNFα, and BDNF from microglia.** Isolated rat microglial cultures were treated with 4 mM mβcd for 30 min, washed and allowed to rest 30 min, then treated with 1 mM hpβcd-cholesterol for 30 min, washed and allowed to rest 30 min. Cultures were activated with 1 μg/ml LPS overnight (18 h) and media was collected and assayed for release of Il1β **(A)**, TNFα **(B)**, and BDNF **(C)**. All analyses were normalized to the total protein of the corresponding cell lysate to control for cell viability. Phagocytosis was assayed after cholesterol depletion **(D-E)** as in Figure 3. Dagger represents P <0.05 by Mann–Whitney U-test while asterisks represent P <0.05, 0.01, and 0.001 by 2-way ANOVA with Bonferroni’s post-hoc from N = 3 independent preparations.

Given the observed sensitivity of microglial cytokine and trophic factor release to changes in cholesterol induced pharmacologically with simvastatin treatment, we sought to examine the effects of direct manipulation of cellular cholesterol levels using methyl-β-cyclodextrin (mβcd), a water soluble reagent capable of removing and ‘chelating’ cholesterol from the plasma membrane. In contrast with simvastatin treatment, which requires sustained treatment to induce decreases in cellular cholesterol levels, acute treatment with 4 mM mβcd decreased cellular cholesterol rapidly, resulting in a 19.8 ± 5.4% reduction within 30 minutes. As with simvastatin treatment, the concentration of mβcd was titrated in initial experiments to achieve consistent changes in cellular cholesterol level without adverse effects on viability. Contrary to our findings with simvastatin, treatment with 4 mM mβcd significantly increased IL1β and BDNF release from unstimulated microglia (Figure 4A,C). This increased release of Il1β was similarly observed in cultures treated with mβcd and subsequently activated with LPS while a comparable, but not significant trend towards increase in BDNF release was observed following LPS treatment (Figure 4A, C). No significant changes were observed in the release of TNFα without LPS activation, while TNFα release from LPS activated microglia was decreased in the mβcd treated group (Figure 4B). As with simvastatin treatment, no significant changes were observed for NO release (data not shown) from mβcd treated microglia with or without LPS-activation. To determine the specificity of these effects, microglia were treated with hpβcd-cholesterol to restore cholesterol back to the plasma membrane. Surprisingly, treatment with hpβcd-cholesterol after mβcd treatment decreased, but did not restore Il1β release to baseline in unstimulated microglia, yet markedly decreased Il1β release from LPS-activated microglia (Figure 4A). As was observed in the simvastatin treated groups, cholesterol decreased Il1β release from LPS-activated microglia independent of mβcd treatment, in both the mβcd-treated and control groups. In contrast, hpβcd-cholesterol treatment did not affect TNFα release in any of the conditions tested (Figure 4B), but was able to attenuate the release of BDNF from both unstimulated and LPS activated mβcd-treated microglia (Figure 4C). The failure of cholesterol to restore TNFα release in mβcd- and LPS-treated microglia suggests it may be a secondary effect of the treatment not attributable to the changes in cholesterol level. The effects of mβcd on BDNF and Il1β are responsive to cholesterol level, though as Il1β release from LPS-activated microglia was attenuated by cholesterol even in the absence of prior mβcd treatment, we infer that Il1β is particularly sensitive to cellular cholesterol levels, as treatment with hpβcd-cholesterol resulted in an increase in mean cellular cholesterol of 77.8 ± 13.9% above control.

Interestingly, treatment of microglia with mβcd also resulted in a significant reduction in the proportion of active phagocytes and the mean uptake of latex beads per phagocyte specifically in cells activated with LPS (Figure 4D-E). Subsequent recovery of cholesterol by hpβcd-cholesterol treatment restored phagocytosis to that of control groups for both proportion of active phagocytes and mean uptake per cell. Unlike Il1β release, supplementing cholesterol in control groups by hpβcd-cholesterol treatment did not have any significant effect on phagocytosis.

## Discussion

In rat primary microglial cultures, statin treatment resulted in reduced secretion of Il1β, and increased secretion of TNFα and BDNF from activated microglia. The altered release profile of microglia was largely reversed by treatment with exogenous cholesterol, suggesting a cholesterol-dependent mechanism. Treatment with mβcd, a cholesterol depleting reagent, increased the release of Il1β and BDNF from microglia and inhibited phagocytosis in LPS-activated microglia in a manner that was reversed by the addition of exogenous cholesterol. Conversely, simvastatin treatment inhibited phagocytosis in a cholesterol-independent manner, being rescued only by MEV but not cholesterol. The disparity in the effects of simvastatin on molecular and functional outputs suggests that changes in microglial activity after statin treatment are the result of two distinct and separable molecular mechanisms.

### Statin treatment alters the release profile of microglia

Treatment of isolated microglia resulted in differential effects on many of the released effectors, in a manner which could alternately be interpreted as either inflammatory or trophic. The reduction in Il1β secretion, generally considered to be a pro-inflammatory cytokine, after simvastatin treatment in ramified or surveilling, but not activated, microglia (Figure 1D) can be interpreted as anti-inflammatory, and the observed increase in BDNF release (a potent neurotrophin and neuroprotectant) from LPS-activated cells after simvastatin treatment would likely be trophic in the brain. Similar reductions in Il1β were observed after lovastatin treatment on rat primary microglia [40] and *in vivo* in a rat model of simvastatin treatment following traumatic brain injury [37], though a recent study found simvastatin treatment increased secretion of Il1β from isolated primate microglia [42]. In contrast, TNFα secretion is increased in simvastatin treated microglia in a cholesterol-dependent manner. TNFα signalling through CD120 can result in toxic effects by downstream activation of apoptosis through caspase 8 cleavage, or survival effects by anti-apoptotic activation through nuclear factor κ-B (NFκB) and c-Jun N-terminal kinase (JNK) signalling. In this regard increased secretion of TNFα from activated microglia may either promote neuronal survival or contribute to neurotoxicity. Several studies reported increases in TNFα levels following statin treatment both *in vitro* and *in vivo*: TNFα levels increased with simvastatin treatment of cultured primate microglia [42], simvastatin or mevastatin treatment increased TNFα levels in *ex vivo* hippocampal slices [43], and TNFα levels were increased in perilesional tissue following TBI and simvastatin treatment [37]. Comparable increases in TNFα were seen in human blood derived macrophage cultures treated with simvastatin [24]. To our knowledge this is the first study to demonstrate increased release of BDNF from LPS-activated microglia following simvastatin treatment.

### Simvastatin affects secretion through cholesterol-dependent mechanisms

Following simvastatin treatment, most of the observed changes in secretory profile were reversed by directly supplementing microglia with cholesterol. The increased release of both TNFα and BDNF from LPS-activated, simvastatin treated microglia was reversed by treatment with hpβcd-cholesterol, returning mean cellular cholesterol to levels at or above that of controls (Figure 1A, B). TNFα release from simvastatin-treated microglia was also attenuated by treatment with MEV, which is expected as mevalonic acid is the immediate product of HMG-CoA reductase and acts as the precursor to both sterol and isoprenoid biosynthesis. These changes strongly suggest that simvastatin affects microglial secretion through a cholesterol-dependent mechanism, rather than through a mechanism mediated by isoprenoids. Two prior studies offer conflicting interpretations regarding this mechanism: in primate microglial cultures TNFα release was found to be cholesterol-dependent [42]; in rat hippocampal slices TNFα levels reverted to control after MEV treatment or treatment with geranylgeranyl pyrophosphate, the first committed intermediate in isoprenoid biosynthesis [43], though a role for cholesterol was not directly tested, the *ex vivo* system precludes resolution of cell-specific effects. In a comparable study on peripheral immune cells, an observed increase in TNFα release from simvastatin treated human blood derived macrophages was reversed by MEV treatment, but as in the aforementioned study, a direct role of cholesterol was not tested [24]. As release of NO from LPS-activated microglia was unaffected by any of the treatments used in this study (Figure 1C and further data not shown), we can speculate that neither simvastatin treatment nor cholesterol-depletion with mβcd affect activation of microglia through Toll-like receptor 4 (TLR4) mediated activation of NFκB, as such a mechanism would be expected to affect the expression of inducible nitric oxide synthetase (iNOS).

To our knowledge, this is the first report to demonstrate changes in Il1β secretion from primary microglia following direct manipulation of cholesterol, though a comparable increase in Il1β secretion was observed after mβcd treatment of the immortalized microglial BV-2 cell line [48]. In our assays an inverse correlation was observed between directly manipulated cholesterol levels and Il1β release: decreasing cholesterol resulted in increased Il1β release, while increasing cholesterol markedly decreased Il1β release even after stimulation with the potent TLR4 agonist, LPS (Figures 1D and 4A). Curiously, while decreasing cholesterol through statin treatment resulted in lower Il1β levels (Figure 1D), directly decreasing cellular cholesterol content through mβcd treatment resulted in an increase in Il1β with or without LPS-activation (Figure 4A). This discrepancy may be explained by one of two models. First, Il1β release may be affected by two distinct mechanisms – one sensitive to cholesterol in the plasma membrane, and one sensitive to intracellular cholesterol. Simvastatin acts on HMG-CoA reductase, an enzyme localized primarily to the endoplasmic reticulum [49], and so reduces cholesterol from the ‘inside-out’ , likely resulting in changes in cholesterol in intracellular compartments followed by later changes in plasma membrane cholesterol. Conversely, mβcd acts directly on the extracellular leaflet of the plasma membrane (PM), reducing cholesterol from the ‘outside-in’ , rapidly affecting PM cholesterol, which subsequently equilibrates across membrane leaflets rapidly, but is then transported into intracellular compartments by relatively slow trafficking through vesicles or carrier proteins. A second interpretation of these data would require a distinct isoprenoid-dependent mechanism affecting Il1β release from statin-treated microglia. In either scenario the observed decreased release of Il1β from simvastatin-treated, LPS-activated microglia after hpβcd-cholesterol treatment may represent the PM cholesterol-dependent mechanism masking the contribution of subtler cholesterol- or isoprenoid-dependent mechanisms. The sensitivity to PM cholesterol is consistent with observations that, unlike TNFα and BDNF, Il1β secretion is dependent on the non-canonical pathway of cytokine secretion. TNFα and BDNF are trafficked in conventional ER- and Golgi-derived vesicles (as a membrane bound pro-cytokine in the case of TNFα and in dense-core vesicles for BDNF) while Il1β is synthesized in the cytoplasm and is secreted either though budding of microvesicles from the PM or possible transport through specific PM resident transporters (eloquently reviewed in [50]). This non-canonical secretory pathway is likely to be differentially sensitive to the lipid composition of the PM than conventional Golgi trafficking, as cholesterol affects membrane fluidity and curvature (as required for vesicular budding) [51–53] as well as regulating protein activity through lipid microdomains (as may affect membrane resident transporters) [54–56]. As such, we propose that the observed sensitivity of Il1β release to cholesterol, in particular the marked decrease in Il1β release from LPS-activated microglia treated with hpβcd-cholesterol (Figure 1D, 4A), may result from a blockade of the late release steps of Il1β. However, as a detailed mechanistic analysis of Il1β secretion was not the objective of this study further analysis will be explored in future works. The increase in basal secretion in the simvastatin control groups can be attributed to the use of an ethanolic vehicle for delivery of simvastatin. As ethanol is itself a TLR4 agonist [57,58] even at the very low ethanol concentrations used (<0.005%), the vehicle resulted in slightly higher Il1β secretion as compared to an untreated control (compare Figures 1D, 2, 3 and Figure 4A). We do not consider this to be a significant confound as the vehicle effect was slight, relative to potent TLR4 activation by LPS, and was not found to affect any of the other measures reported (TNFα, BDNF, NO, viability, or phagocytosis).

### Simvastatin and mβcd inhibit phagocytosis through distinct mechanisms

Both cholesterol lowering treatments tested in this study reversibly inhibited phagocytosis of fluorescent latex beads. Treatment of microglia with 2 mM mβcd significantly reduced both the proportion of active phagocytes and the mean uptake per phagocyte, both of which were fully recovered after treatment with hpβcd-cholesterol (Figure 4D-E). Simvastatin treatment comparably reduced both measures but phagocytosis was only recovered with MEV, but not hpβcd-cholesterol treatment (Figure 3A-B), suggesting a cholesterol-independent mechanism. To our knowledge, this is the first report of the effects of statins on phagocytosis in primary microglia, though a study using the BV-2 cell line demonstrated inhibition of amyloid-β induced phagocytosis after simvastatin and lovastatin treatment [59]. Comparable studies of phagocytosis in cultured macrophages have shown statin treatment was inhibitory in human blood derived monocytes and macrophages [24,26], rodent peritoneal macrophages [25], and in the immortalized THP-1 macrophage cell line [60]. Kuipers and colleagues [41] demonstrated inhibitory effects of statin treatment on cytoskeletal dynamics and motility in primary microglial cultures – both of which would contribute to phagocytic mechanisms, but stopped short of testing phagocytosis. Others report enhancement of phagocytosis after statin treatment of rodent peritoneal macrophages [28,29] and human blood derived monocytes [27]. Reports are likewise mixed as to the mechanism of statin mediated inhibition of phagocytosis, with two showing rescue with cholesterol [25,28], two with MEV alone [41,59], and one with MEV but not squalene, the first committed intermediate in cholesterol synthesis [24]. Our data suggest that phagocytosis in primary microglia can be inhibited by cholesterol-dependent means (demonstrated by mβcd treatment and recovery with cholesterol, Figure 4D-E) or by cholesterol-independent means (demonstrated by simvastatin treatment and recovery only by MEV, Figure 3A-B). As simvastatin was less effective at depleting cholesterol than mβcd (at the doses we tested) it remains possible higher simvastatin doses would reveal a cholesterol-dependent component, though such treatments may not represent relevant therapeutic dosage [61].

### Separable mechanisms of statin action on microglia

Collectively, these data suggest statin treatment directly affects the secretory profile of microglia and microglial phagocytosis through two distinct mechanisms. Simvastatin-dependent changes in the secretion of cytokines and trophic factors were reversed by the addition of exogenous cholesterol, suggesting a cholesterol-dependent role in release, while simvastatin-dependent changes in phagocytosis were reversed by co-treatment with MEV but not through delivery of exogenous cholesterol, suggesting a cholesterol-independent role in phagocytosis. This represents the first coupled analysis of both the molecular and functional changes associated with statin treatment on primary cultured microglia. Interestingly we can uncouple these two effects through selective rescue, as simvastatin and cholesterol treatment resulted in cells with ‘normal’ release of TNFα and BDNF that remained phagocytosis-deficient. These results may also help to explain conflicting reports on the effects of statin treatment on cultured microglia and macrophages – different markers of activation and secreted factors may be influenced by distinct signalling pathways with the same treatment. While the field remains unsettled as to the mechanistic contributions of cholesterol-dependent and independent components of statin therapy on pathological conditions of the CNS, these data suggest there remains room to improve the specificity of pharmacotherapy. Ultimately, improved pharmacological agents will target only those pathways that have the most direct impact on the dysfunction of microglia in neuroinflammatory conditions.

## Materials and methods

### Materials

Dulbecco’s modified Eagle Medium/Ham’s F-12 (DMEM/F-12, 1:1) was from Hyclone (Thermo-Fisher Scientific, Ottawa, ON). Fetal bovine serum (FBS), normal horse serum (NHS), penicillin/streptomycin, 0.25% trypsin/EDTA, and Hank’s balanced saline solution (HBSS) were from Gibco (Life Technologies, Burlington, ON). Simvastatin was from Calbiochem (EMD Millipore, Billerica, MA). Mevalonolactone, methyl-β-cyclodextrin, and 2-hydroxypropyl-β-cyclodextrin were from Sigma (Oakville, ON). VectaShield mounting medium with 4’, 6-diamidino-2-phenylindole (DAPI) was from Vector Labs (Burlington, ON). All other reagents were of the highest quality available.

### Primary microglial cell culture and treatments

All animal procedures were carried out in accordance with the guidelines of the University of Alberta Animal Care Committee. Microglia were isolated from mixed glial cultures at 14–21 days *in vitro* (d.*i.v*.) according to the method of Saura [62,63]. In brief, whole brains were dissected from postnatal day one Sprague–Dawley rat pups, meninges removed, and cells dissociated by trypsinization and trituration. Mixed cultures were grown in 12-well plates coated with poly-L-lysine and maintained in DMEM/F-12 with 10% FBS and 200 U/ml penicillin, 200 μg/ml streptomycin in a 37°C, 5% CO_2_ humidified incubator. After reaching confluence, microglia were isolated by mild trypsinization (0.25% trypsin/EDTA diluted to 30% strength with DMEM/F12) for 20 minutes. Isolated cultures were maintained in DMEM/F12 with 1% FBS and 200 U/ml penicillin, 200 μg/ml streptomycin for the duration of the experiment. Purity of microglial isolations was routinely assessed by immunofluorescence microscopy for microglia-specific markers (Iba1 or CD68) compared to total nuclear staining with DAPI and was consistently ≥98% microglia.

Treatments were carried out 24 h after isolation. Simvastatin was first hydrolysed from the stable lactone to the bioactive free acid in alkali ethanol followed by neutralization with 1 N HCl. Mevalonolactone (MEV, the lactone isomer of mevalonic acid) was delivered from aqueous preparations. Cholesterol was delivered using 2-hydroxypropyl-β-cyclodextrin as vehicle [52,64]. All treatments were carried out against an appropriate vehicle control.

### Molecular analyses

Nitric oxide levels were estimated by measuring the major metabolite nitrite using the Griess reaction [65,66]. Cholesterol was assayed using an enzymatic Amplex Red assay (Life Technologies, Burlington, ON) according to the manufacturer’s instructions.

Enzyme-linked immunosorbant assays (ELISA) for TNFα and Il1β were performed according to the manufacturer’s instructions (R&D Systems, Minneapolis, MN), while BDNF was analysed by competitive ELISA. Briefly, media samples or BDNF standards in 96-well plates were incubated in coating buffer (100 mM Na_2_CO_3_, 10 mM NaHCO_3_, pH 9.6) with goat anti-BDNF antibody (1:100, Santa Cruz Biotechnology, Dallas, TX) overnight at 4°C. Plates were washed 3x with phosphate buffered saline +0.05% Tween-20 (PBS-T) and blocked with 1% BSA in PBS for 2 h at room temperature. After washing, plates were incubated with a biotin-conjugated anti-goat secondary antibody (1:2000 in PBS +1% BSA) for 1 h, followed by horseradish peroxidase-conjugated streptavidin (1:200 in PBS +1% BSA) for 20 minutes. Colour was developed by adding tetramethylbenzidine (0.027% in 100 mM sodium acetate, 19 mM citric acid, 40% methanol, and 0.3% H_2_O_2_) for 20 minutes at room temperature. Assay development was stopped by the addition of 1.8 N H_2_SO_4_ and measured at 450 nm with correction at 570 nm.

Viability was routinely assessed for all treatment conditions by incubation with 0.5 mg/ml 3-(4,5-dimethylthiazol-2-yl)-2,5-diphenyltetrazolium bromide (MTT) for 30 min and measurement of the oxidized formazan end-product at 540 nm.

All molecular analyses were normalized to the protein level of the cell lysate, measured using the BCA assay (Thermo-Fisher Pierce, Ottawa, ON) to account for any variability in cell numbers between wells.

### Immunocytochemistry

At the end of treatment, cells were washed 3x with HBSS supplemented with 1 mM Ca^2+^ and 0.5 mM Mg^2+^ and fixed in 5% PBS-buffered formalin for 10 minutes at room temperature. Each of the following sequential steps was preceded by washing 3x with PBS: fixed cells were blocked and permeablized (PBS supplemented with 1% NHS and 0.1% triton X-100 for 1 h), incubated with primary antibody (dilutions as indicated in Table 1 in PBS with 0.1% NHS, overnight at 4°C), incubated with Alexa conjugated secondary antibody (dilutions as indicated in Table 1 in PBS with 0.1% NHS, 2 h at room temperature), and mounted using Vectashield with DAPI. Images were acquired using a Leica AF6000-LX microscope. Confocal microscopy was performed on a Leica TCS-SPE inverted microscope. Post-processing was performed using ImageJ.

**Table 1:** **Antibodies used for immunocytochemistry**

**Antigen**	**Host**	**Manufacturer**	**Dilution**
**Ionized Ca** ^**2+**^ **-binding protein 1 (Iba1)**	Rabbit	Wako	1:1000
**CD68 (ED-1 clone)**	Mouse	Serotec	1:750
**Glial fibrillary acidic protein (GFAP)**	Mouse	Sigma	1:1000
**GFAP**	Rabbit	Dako	1:1000
**Mouse-Alexa Fluor 488 conjugate**	Donkey	Life Technologies	1:500
**Rabbit-Alexa Fluor 488 conjugate**	Donkey	Life Technologies	1:500
**Mouse-Alexa Fluor 647 conjugate**	Donkey	Life Technologies	1:500
**Rabbit-Alexa Fluor 647 conjugate**	Donkey	Life Technologies	1:500

### Phagocytosis assay

Phagocytosis was assayed by incubating treated cells with 1x10^7^ green fluorescent 1 μm carboxylate-modified latex beads (Sigma, Oakville, ON) per well for 3 h. Cells were then processed for immunocytochemistry as above with CD68 as a marker for microglia and DAPI as a nuclear stain. Images were acquired according to a defined grid (21 images per well) to reduce user bias and analyzed automatically using macros custom written for ImageJ. Briefly, total cells were identified by nuclear staining and a region of interest (ROI) was defined to enclose the cell soma. Fluorescence was integrated for green (GFP filters, beads) and red channels (CY5 filters, CD68 immunofluorescence) within the somatic ROI. CD68 immunofluorescence was used to validate culture purity. A subset of images was randomly selected to manually validate the results of automated analysis. Total phagocytes are reported as the proportion of cells per well that took up at least 1 bead, while cellular phagocytic activity was defined as the mean integrated fluorescence of cells that had taken up at least 1 bead.

### Statistical analyses

Overall significance was assessed using 2-way analysis of variance, with Bonferroni’s multiple comparison post-hoc analysis between groups. Pairwise comparisons were assessed using a Mann–Whitney U test. A P value of ≤0.05 was considered significant. Data are presented as the mean ± SEM. Each reported N indicates an independent experiment from a separate culture preparation.

## Abbreviations

BDNF: Brain derived neurotrophic factor
CNS: Central nervous system
DAPI: 4',6-diamidino-2-phenylindole
ELISA: Enzyme-linked immunosorbant assay
FBS: Fetal bovine serum
HMG-CoA: 3-hydroxy-3-methylglutaryl-coenzyme A
Hpβcd: 2-hydroxypropyl-β-cyclodextrin
HBSS: Hank’s balanced saline solution
Iba1: Ionized Ca^2+^-binding protein 1
Il1β: Interleukin-1β
JNK: c-Jun N-terminal kinase
LPS: Lipopolysaccharide
mβcd: Methyl-β-cyclodextrin
MEV: Mevalonolactone/mevalonic acid
MHCII: Major histocompatibility complex II
MTT: 3-(4,5-dimethylthiazol-2-yl)-2,5-diphenyltetrazolium bromide
NFκB: Nuclear-factor κB
NHS: Normal horse serum
NO: Nitric oxide
PM: Plasma membrane
TLR4: Toll-like receptor-4
TNFα: Tumour necrosis factor-α
